# Tissue inhibitor of metalloproteinases 1 enhances rod survival in the *rd1* mouse retina

**DOI:** 10.1371/journal.pone.0197322

**Published:** 2018-05-09

**Authors:** Hwa Sun Kim, Andrew Vargas, Yun Sung Eom, Justin Li, Kyra L. Yamamoto, Cheryl Mae Craft, Eun-Jin Lee

**Affiliations:** 1 MDA Vision Research, USC Roski Eye Institute, Department of Ophthalmology, Keck School of Medicine of the University of Southern California, Los Angeles, California, United States of America; 2 Department of Integrative Anatomical Sciences, Keck School of Medicine of the University of Southern California, Los Angeles, California, United States of America; 3 Department of Biomedical Engineering, Viterbi School of Engineering, University of Southern California, Los Angeles, California, United States of America; University of Massachusetts Medical School, UNITED STATES

## Abstract

Retinitis pigmentosa (RP), an inherited retinal degenerative disease, is characterized by a progressive loss of rod photoreceptors followed by loss of cone photoreceptors. Previously, when tissue inhibitor of metalloproteinase 1 (TIMP1), a key extracellular matrix (ECM) regulator that binds to and inhibits activation of Matrix metallopeptidase 9 (MMP9) was intravitreal injected into eyes of a transgenic rhodopsin rat model of RP, S334ter-line3, we discovered cone outer segments are partially protected. In parallel, we reported that a specific MMP9 and MMP2 inhibitor, SB-3CT, interferes with mechanisms leading to rod photoreceptor cell death in an MMP9 dependent manner. Here, we extend our initial rat studies to examine the potential of TIMP1 as a treatment in retinal degeneration by investigating neuroprotective effects in a classic mouse retinal degeneration model, *rd*^*Pde6b-/-*^ (*rd1*). The results clearly demonstrate that intravitreal injections of TIMP1 produce extended protection to delay rod photoreceptor cell death. The mean total number of rods in whole-mount retinas was significantly greater in TIMP-treated *rd1* retinas (postnatal (P) 30, P35 (P<0.0001) and P45 (P<0.05) than in saline-treated *rd1* retinas. In contrast, SB-3CT did not delay rod cell death, leading us to further investigate alternative pathways that do not involve MMPs. In addition to inducing phosphorylated ERK1/2, TIMP1 significantly reduces BAX activity and delays attenuation of the outer nuclear layer (ONL). Physiological responses using scotopic electroretinograms (ERG) reveal b-wave amplitudes from TIMP1-treated retinas are significantly greater than from saline-treated *rd1* retinas (P<0.05). In later degenerative stages of *rd1* retinas, photopic b-wave amplitudes from TIMP1-treated *rd1* retinas are significantly larger than from saline-treated *rd1* retinas (P<0.05). Our findings demonstrate that TIMP1 delays photoreceptor cell death. Furthermore, this study provides new insights into how TIMP1 works in the mouse animal model of RP.

## Introduction

The cell-extracellular matrix (ECM) interaction influences cell survival by regulating gene expression, differentiation, and growth [1]. The integrity of the ECM in retina requires the balance between matrix metalloproteinases (MMPs) and the tissue inhibitors of metalloproteinases (TIMPs) [2–4]. Working together, these players regulate neural organization by remodeling ECM in normal and pathologic retinas [5, 6]. Recently, TIMPs were discovered to also be involved in apoptosis regulation [7]. Among members of the TIMP family, TIMP1 inhibits apoptosis in various cell types and conditions, including neurons in the hippocampus in the seizure model [8], human primary cultured neurons with HIV-1 induced inflammation [9], and mammary epithelial cells in transgenic mice overexpressing TIMP1 [10]. Additionally, TIMP1 is significantly upregulated in human and animal models with various ocular diseases including retinal degeneration [11–13], indicating that TIMP1 may have a critical role against intrinsic apoptotic cell death [7, 14]. Although TIMP1 has been reported to inhibit apoptosis of several types of cells, it is not definitive that the inhibition of cell death must depend on its ability to inhibit MMPs [15, 16]. When the MMP-inhibitory domain of TIMP1 is mutated, the anti-apoptotic effects were no longer produced in hepatic stellate cells, suggesting that the inhibition of apoptosis is dependent on MMPs inhibitory activity [17]. In contrast, other reports clearly show anti-apoptotic effects by TIMP1 were independent of its inhibitory activity on MMPs in different cellular systems such as ischemic brain, glutamate excitotoxicity-induced hippocampal cells, Burkitt’s lymphoma, and kidney epithelial cells [18–21]. TIMPs contain both structurally and functionally distinct N- and C-terminal domains [22]. The N-terminal domain of TIMPs contains stable native structures and is fully active as inhibitors of MMPs and some disintegrin-metalloproteinases (ADAMs and ADAMTSs) [23–27]. In addition, the N-domain has residues that interact with the Zn^2+^ binding site of active MMPs [28]. In contrast, the C-terminal domain of TIMPs activates cell survival in an MMP-independent manner through cell-signaling pathways [20, 21, 29]. Interestingly, TIMP1, through its C-terminal domain, has been linked to regulation of specific cell-signaling pathways to promote cellular growth and inhibit apoptosis [15, 16, 30, 31]. TIMP1 inhibition of apoptosis in various cells involves focal adhesion kinase (FAK) and mitogen-activated protein kinase (MAPK)-mediated cell survival signaling via cell surface receptors, CD63 and 1 integrin [27, 28], rather than regulation of cell interactions with ECM through MMP inhibitory activity [32, 33].

In our previous study, TIMP1 treatment partially protected retinal cone outer segments in a rat transgenic rhodopsin model of Retinitis Pigmentosa (RP), S334ter-line3, implicating that TIMP1 plays a role as a survival factor in RP retina [34]. In this current study, we examine the neuroprotective potential of TIMP1 in photoreceptors in mouse *rd*^*Pde6b-/-*^ (*rd1*) retina. We show that TIMP1 significantly reduced BAX activity and delayed thinning of the outer nuclear layer (ONL) in earlier retinal degenerative stages. Furthermore, our results demonstrate that TIMP1-mediated rod survival is MMP9-independent, at least in part, works through an ERK survival pathway in mouse *rd*^*Pde6b-/-*^ (*rd1*) retina. Thus, TIMP1 delays photoreceptor cell death via mechanisms other than MMP9 inhibitory activity in RP retina. These data suggest a novel neuroprotective role of TIMP1 and provide insight into designing mutation-independent treatment.

## Materials and methods

### Animals

All animals were treated with approved protocols by the Association for Research in Vision and Ophthalmology Statement for the Use of Animals in Ophthalmic and Vision Research and with the regulations of the Veterinary Authority of the University of Southern California. The homozygous *rd1* (retinal degeneration 1, *Pde6b*^*rd1*^), the first generation of retinal degeneration with a mutation in exon 7 of the *Pde6b* gene encoding beta subunit of cyclic guanosine monophosphate—phosphodiesterase (cGMP-PDE), on a C57BL / 6J background mice were used [35, 36]. Female or male *rd1* mice were euthanized at postnatal (P) days 10, 14, 15, 16, 17, 18, 30, 35, 45, 60, and 90 (number (n) = 9–12, respectively for each stage). For normal C57BL ⁄ 6J black mice (P10, P14, P18, P30, P45, P60, and P90) (The Jackson Laboratory, Bar Harbor, ME, USA) were used (n = 5–7, for each stage). For all experiments, animals were housed in cyclic 12-hour light/dark conditions with free access to food and water.

### Administration of TIMP1 and SB3-CT

The procedure on the preparation of TIMP1 (Sigma-Aldrich Corp., St. Louis, MO, USA) and SB-3CT (-[(4-phenoxyphenyl) sulfonylmethyl] thiirane, EMD Millipore, Temecula, CA, USA) were similar with our previous study [34, 37, 38]. 1 μl (25 μg/ml) of TIMP1 in phosphate buffered saline (PBS) was administered by intravitreal injection with a Hamilton syringe (33 gauge needle, Sigma-Aldrich Corp.). For each animal, the left eye was intravitreally injected with TIMP1 and the right eye was injected with saline (1 μl) for comparison. Injection procedures of SB-3CT were identical as the TIMP1 injection. For each animal, the left eye was injected with 1 μl of SB-3CT (25 μg/ml) in PBS with 0.05–0.1% dimethyl sulfoxide (DMSO) [39] and the right eye was injected with PBS with 0.05–0.1% DMSO for comparison. The developmental stage for the injection of SB-3CT or TIMP1 was either P15 (i.e. time period of peak rod death and time of eye opening) [36], or P45, when the majority of rod photoreceptor cells were degenerated (see Results). Surgeries on mice were performed under anesthesia induced by intraperitoneal injection of ketamine (20 mg/kg; KETASET, Fort Dodge, IA, USA) and xylazine (5 mg/kg, X-Ject SA; Butler, Dublin, OH, USA). Following intravitreal injection, veterinary ophthalmic antibacterial ointment was applied to prevent drying of cornea and infection. For all experiments, TIMP1, SB-3CT, and saline were injected at the same time of the day, Zeitgeber Time (ZT) 4 (ZT 0 defined as the moment lights were turned on), and tissues were isolated at each respective time point [40]. For all experiments, animals were housed in cyclic 12-hour light/dark conditions.

### Tissue preparation & immunohistochemistry

Detailed protocols for tissue preparation were described and published [37, 38]. Briefly, animals were anesthetized by IP injection of Euthasol (40 mg/kg; Virbac Corporation, Fort Worth, TX) and the eyes were enucleated for the collection of retinal tissue. Then, animals were euthanized by administration of an overdose of Euthasol. For a secondary method, we performed thoracotomy or decpitations. Eyecups were fixed with 4% paraformaldehyde in 0.1 M phosphate buffer (PB), for 90 minutes at 4°C after removing the anterior segment and lens. Following fixation, we isolated the retinas from eye cups for whole mount staining, and then incubated retinal tissues with 10% normal donkey serum (NDS) (#017-000-121, Jackson ImmunoResearch Laboratories, West Grove, Pennsylvania, dilution 1:10) for 1 hour at room temperature (RT), then incubated overnight with primary antibodies: mouse monoclonal antibody directed against rhodopsin (rho 1D4, dilution 1:1,000 [41]), and rabbit polyclonal antibodies M-opsin (dilution 1:1,000) [42]. Then tissues were washed three times for 10 minutes each with PBS, and incubated for 1 day at RT in corresponding secondary antibodies with carboxymethylindocyanine (Cy3)-conjugated affinity-purified donkey anti-rabbit IgG (Jackson ImmunoResearch Laboratories, dilution 1:500) or Alexa 488-conjugated donkey anti-mouse IgG (Molecular Probes, Eugene, OR, USA; dilution 1:300). Next, tissues were washed three times for 10 minutes each with PB and cover-slipped with Vectashield mounting medium (Vector Labs, Burlingame, CA). The images of whole mounts were saved and processed with the Zeiss LSM-PC software under a Zeiss LSM 710 confocal microscope (Zeiss, NY), then each stained rod cell body was marked with a dot using the paint tool in Photoshop (Adobe Systems, San Jose, CA) to generate retinal maps of each retina.

### Immunoblot analysis

For immunoblot analysis, whole retinal cell lysate was obtained from each frozen retina following homogenization in ice-cold Radioimmunoprecipitation assay (RIPA) buffer, supplemented with EDTA-free proteinase inhibitor cocktail (Roche, Basel, Switzerland). Following homogenization, samples were centrifuged at 16,000 g for 10 min, and the supernatant was collected to measure protein concentrations using Pierce BCA assay kit (Thermo Scientific, Rockford, IL). 50 μg of retinal extracts were applied to electrophorese on a 10% sodium dodecyl sulfate-polyacrylamide gel (SDS-PAGE), and then were transferred to nitrocellulose membranes (LI-COR Biotechnology, Lincoln, NE). After 1 hour of blocking with Odyssey blocking buffer (LI-COR Biotechnology), membranes were incubated overnight with mouse monoclonal antibodies for anti-β-actin (#A5441, Sigma, dilution 1:5,000) and either rabbit polyclonal anti-phosphorylated Extracellular Signal-regulated Kinase (pERK) 1/2 (#9101S, Cell Signaling, dilution 1:500), anti-ERK (#4695S, Cell Signaling, dilution 1:500), anti-phosphorylated Protein Kinase B (pAKT) (#4060S, Cell Signaling, dilution 1:500), anti-AKT (#4691S, Cell Signaling, dilution 1:500), and anti-BAX (#14796S, Cell Signaling, dilution 1:500). Afterwards, appropriate secondary antibodies conjugated to a fluorophore (680 nm or 800 nm) were used for detection using an infrared detection system (GENESys, Syngene, Frederick, MD). For all optical density analysis, we used National Institute of Health (NIH) Image J software version 1.50i to quantify the intensity of each band. β-actin was used as a loading control. Relative amounts of the immunoreactive pERK 1/2, pAKT, and BAX were calculated by dividing the intensity of these proteins by the intensity of the immunoreactive β-actin protein. The saline-treated *rd1* at 5 min was set as 100%.

### Gelatin zymography

Previously, detailed protocols for gelatin zymography were published [38]. 50 μg of retinal protein extracts were mixed with SDS Sample buffer excluding β-mercaptoethanol and boiling and then applied to 10% NOVEX Pre-Cast SDS polyacrylamide gel (Novex Life Technologies) in the presence of 0.1% gelatin under non-reducing conditions for electrophoresis. The 6.6 ng of recombinant mouse/rat MMP2 (R&D Systems, Minneapolis, MN) and recombinant mouse MMP9 (R&D Systems, Minneapolis, MN) were used as positive controls. Following electrophoresis, the zymogram gels were washed with deionized water, and then each was incubated in renaturing buffer for 30 minutes at room temperature and then incubated in developing buffer (Novex Life Technologies) for 30 minutes at room temperature and further for 16 hours at 37°C. After staining with SimpleBlue^™^ Safestain (Novex Life Technologies) for overnight, gels were de-stained in deionized water for overnight and imaged using HP Photosmart 7520 and processed using Photoshop (Adobe, San Jose, CA) software.

### Retinal layer thickness measurement

Eye cups (n = 5) were embedded in Tissue-Tek Optimal Cutting Temperature (OCT) embedding medium (Sakura Finetek, Netherlands) after fixation with 4% paraformaldehyde in 0.1 M PB at 4°C for 90 minutes. Eyecups were sectioned along the vertical meridian on a cryostat at a thickness of 10 μm. TOPRO-3 (Invitrogen, Carlsbad, CA; T3605, dilution 1:1,000) was incubated for 10 minutes and washed for 30 minutes with 0.1M PB and cover-slipped with Vectashield mounting medium. The Zeiss LSM image browser software was used to measure the thickness of outer nuclear layer (ONL) and inner nuclear layer (INL). The measurements were taken within 1 mm from the optic nerve.

### Electroretinography (ERG)

The ERG was performed using the HMsERG system (Ocuscience, Las Vegas, NV, USA) as previously described [43–45]. Briefly, mice were dark-adapted for 12 hours before recordings. Under a dim red illumination, the pupil was dilated with Atropine sulfate ophthalmic solution 1% (Akorn Inc, Lake Forest, IL, USA). The recording electrode attached to the contact lens was placed on the cornea of both eyes. The eye lubricant hypromellose ophthalmic solution, USP 2.5% (HUB pharmaceuticals, LLC, Rancho Cucamonga, CA, USA) was applied to maintain the hydration and conductivity between the cornea and recording electrodes. The reference and ground electrodes were placed subcutaneously. The eyes were then given scotopic ERG responses (a series of white light flashes varying from 100 to 25,000 mcd.s/m^2^). After 10 minutes of light adaptation, photopic ERG responses of 10–25,000 mcd.s/m^2^ were recorded. This program was recommended by the manufacturer for obtaining reliable ERG results on rodents (i.e., a broad-range flash response curve). The amplitudes for the resulting b-wave responses at the light flash intensity of 3,000 mcd.s/m^2^ (i.e., optimal light stimulus for doing ERG recordings in research animals and humans [46]) were plotted in this study.

### Construction of retinal map

Confocal micrographs of the retinas (n = 3 animals for each group) were taken at the focal level of the nuclei of rhodopsin. The micrographs were used to compose collages using Photoshop. Composite images of whole-mount retinas were outlined using the paint tool in Photoshop (S1 Fig). Each nucleus of the immunolabeled rhodopsin rod was visualized using the zoom tool and each nucleus was marked with a white dot using the paint tool in Photoshop. The circular dots were slightly smaller in size of the actual nuclei and were kept even throughout the whole-mount retina. The resulting retinal map allowed easy identification of the position of each rod in the retina (detailed method is described in [37]). The total number of rhodopsin-immunoreactive rods in both *rd1* saline and *rd1* TIMP1 whole-mount retinas were manually counted while putting each dot in composite images of different stages of whole-mount retinas. In addition, the retinal-area for these retinas were also measured by ImageJ (National Institutes of Health, Bethesda, MD).

### Quantification and statistics

Saline-treated, SB-3CT-treated, and TIMP1 treated *rd1* retinas were examined to compare their densities of rhodopsin-immunoreactive rods at P30, P35, and P45. An area of 0.25 mm^2^ in the superior-temporal retina was selected from each retina for measurement. The area was selected because rods in the superior-temporal region remain viable longer than other region of the *rd1* retinas. Briefly, confocal micrographs of the retinas (n = 3 animals for each group) were taken at the mid-region (1 mm away from optic disc) of the superior-temporal retina. At these locations, we made serial optical sections using a confocal microscope. By following each immunoreactive rhodopsin staining positive rod throughout the sections, we ensured that every rhodopsin positive cell in the selected region was counted. All the statistics were expressed as mean ± standard error of the mean (SEM). Student’s t-test was used for comparison (GraphPad Prism 6, La Jolla, CA). Two-way ANOVA and Fisher’s least significant difference procedure (LSD test) were used to examine the differences among the group of means. The statistical tests were performed using GraphPad Prism 6. The difference between the means of separate experimental groups was considered statistically significant at P < 0.05.

## Results

### SB-3CT treatment did not affect the rod survival in *rd1* mouse retina

**Fig 1:**
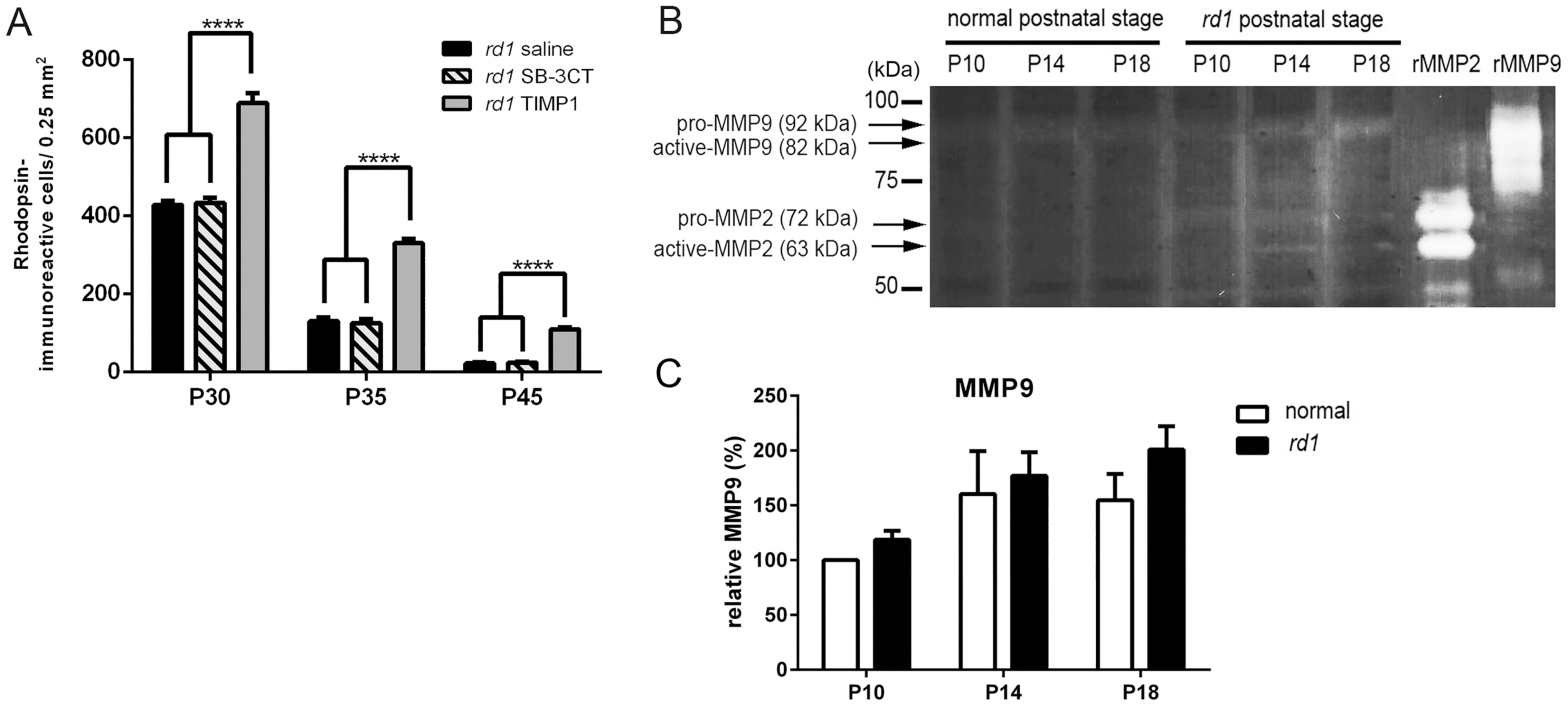
SB-3CT-treatment did not affect the rod survival in the *rd1* mouse retina. The summary graphs illustrate mean rod density measured from the 0.25 mm^2^ sampling areas (in the superior-temporal region) of all saline-treated, SB-3CT-treated, and TIMP1–treated *rd1* retina groups (A). Retinal extracts of normal and *rd1* were collected at P10, P14, and P18 for gelatin zymography (B). Recombinant mouse MMP9 and recombinant mouse/rat MMP2 were applied to the gel and transferred to the membrane as positive controls. Data represent mean ± SEM, ****P<0.0001; P, postnatal.

In our previous report [38], we observed that up-regulation of MMP9 in S334ter-line3 retinas is associated with rod death and SB-3CT, a specific inhibitor of MMP2 and MMP9, dramatically inhibits up-regulated MMP9 expression by interfering with mechanisms that lead to MMP9-dependent apoptosis. Thus, in the current study, we injected either saline, SB-3CT or TIMP1 to P15 *rd1* mice, and then performed rhodopsin immunohistochemical staining on P30, P35, and P45 whole-mount retinas for quantitative analysis to examine the rod cell survival in the *rd1* retina. For each animal, the left eye was injected with TIMP1 or SB-3CT, and the right eye was injected with saline for comparison. Fig 1A shows the mean rod density measured from the 0.25 mm^2^ sampling area (for detail, see Methods) of saline-, SB-3CT-, and TIMP1-treated *rd1* retinas. The mean density of cells in saline-treated *rd1* retinas from TIMP1 and SB-3CT injected animals at each stage was similar. The mean density of cells in saline-treated *rd1* retina at P30, P35, and P45 were 429±9, 130±10, and 23±2 cells/0.25 mm^2^, respectively. The density of cells in SB-3CT treated *rd1* retinas, was similar to saline-treated retinas at P30 (434±12), P35 (126±11), and P45 (24±3) cells/0.25 mm^2^. However, in TIMP1 treated groups, we observed a significant change in the rod cell density. The density of cells in TIMP1 treated *rd1* retina at P30, P35, and P45 showed higher numbers of 689±25, 331±11, and 109±5cells/0.25 mm^2^, respectively. The two-way ANOVA analysis showed significant differences between the means of different groups and the different postnatal days (Fig 1A; **** p < 0.0001).

To determine if the application of SB-3CT treatment (1 μl of 25 μg/ml) did not delay the cell death due to no increases in MMP9 and MMP2 activity in *rd1* retina, we used zymography (Fig 1B). Retinal extracts of normal and *rd1* were collected at P10, P14, and P18. Up-regulation of MMP9 or MMP2 expression was documented in retinal degenerative diseases [47–49]. For example, MMP-9 contributes to excitotoxicity-mediated pathogenesis [48, 50] and neurological disorders [51, 52]. Furthermore, in the *rd1* mouse retina, up-regulation of MMP-9 and MMP-2 has been reported [49]. In our RP model, the degeneration of rods start around P8-10 [36, 53, 54] and shows a dramatic peak between P12-15 [36, 49, 50]. Thus, we have examined the gelatinolytic MMP9 and MMP2 activities at P10 (beginning period of rod death, P14 (peak rod death), and P18 (after peak rod death). Gelatin zymography showed weak gelatinolytic activity of pro- and active-MMP9 in both normal and *rd1* postnatal retinas. The two-way ANOVA analysis showed no significant differences between the mean of normal and *rd1* retinas at P10 (P = 0.56), P14 (P = 0.61), and P18 (P = 0.17) (Fig 1C). However, weak activity of pro-MMP2 (72 kDa) and active-MMP2 (63 kDa) were shown in P10, P14, and P18 *rd1* retinal lysates. We did not detect the gelatinolytic activity of pro-MMP2, and active-MMP2 in normal postnatal retinas (Fig 1B). Recombinant mouse MMP9 and recombinant mouse/rat MMP2 were used as positive controls. Thus, our results suggest that the rod death in *rd1* mouse retina is not linked to MMP9 activity [38]. However, the rod death may have influenced the levels of MMP2 in *rd1* retina. Nevertheless, our data clearly demonstrated that rod cell survival was not protected by SB-3CT treatment. In contrast, TIMP1 treatment substantially enhances survival by delaying rod cell death in *rd1* retina.

### TIMP1 affects rod survival in *rd1* mouse retina

**Fig 2:**
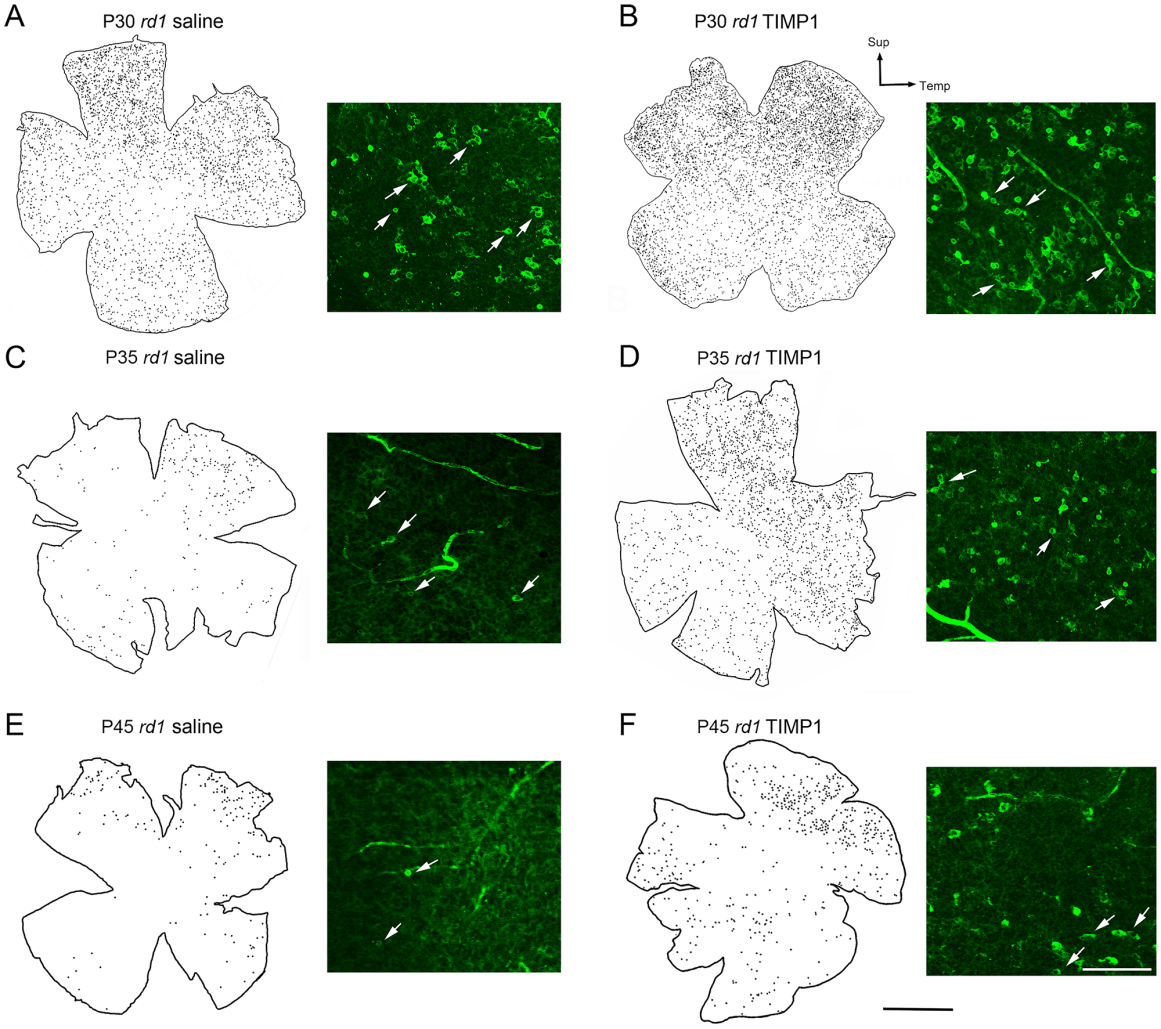
Distribution of survival rods in whole-mount retinas. Distribution of rhodopsin-immunoreactive cells in the outer part of the saline and TIMP1 treated whole-mount retinas of P30, P35, and P45. An example of rhodopsin-immunoreactive cells (arrows) in whole-mount retinas are shown next to retinal map. P, postnatal; Sup, superior; Temp, temporal. Scale bars = 1 mm (applies to all retinal maps), 50 μm (micrographs of rhodopsin immunoreactive cells).

**Fig 3:**
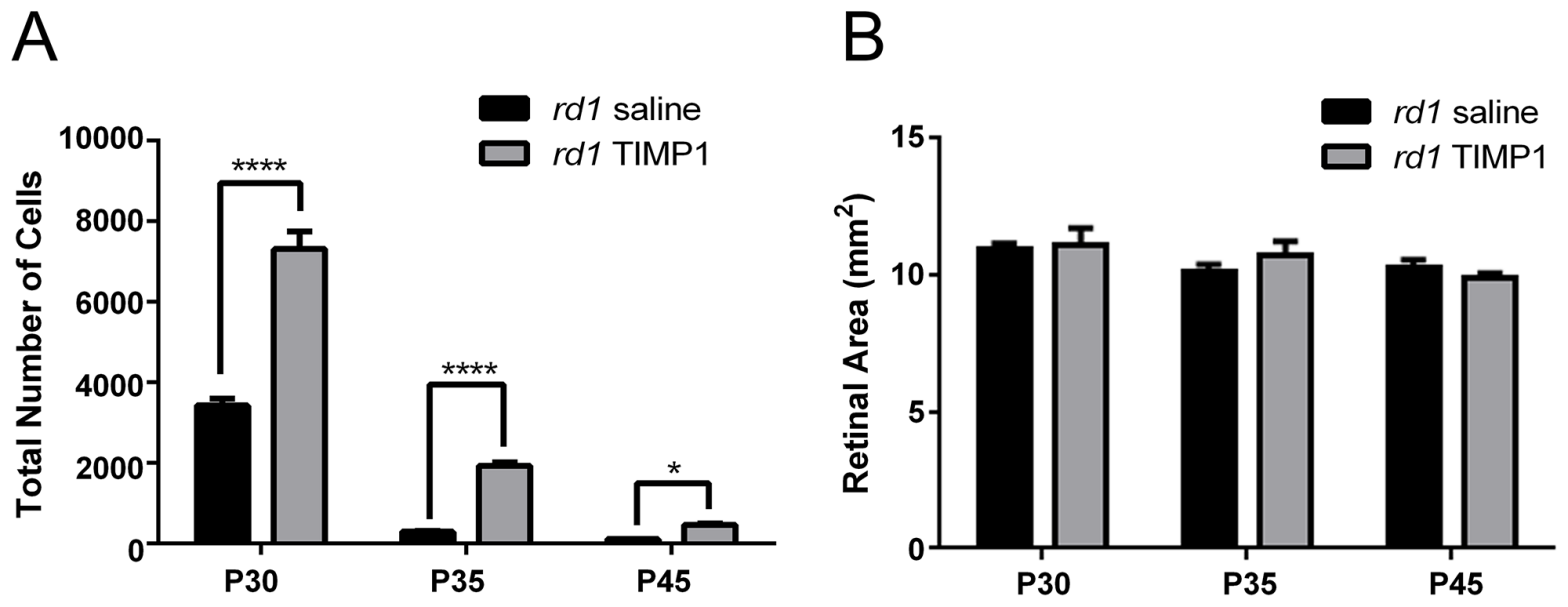
TIMP1 affects rod survival in *rd1* mouse retina. A graph of mean total number of rhodopsin-immunoreactive cells versus postnatal age (A) showed significant differences between the saline-treated and the TIMP1 treated *rd1* retinas at P30, P35, and P45. (B) A graph of saline-treated and TIMP1 treated retinal area (mm^2^) versus postnatal age indicated no significant growth of retina in size with age. There were no significant differences in the retinal area between the saline-treated and TIMP1 treated *rd1* mouse retinas. Data represents mean ± SEM, **** P<0.0001, *P<0.05; P, postnatal.

We further investigated the distribution of rods in saline-treated and TIMP1-treated *rd1* whole-mount retinas at P30, P35, and P45 following a single injection of TIMP1 at P15 (Figs 2 and 3). We began by drawing retinal maps and applying digital dots on rods as shown in Fig 2 (see construction of retinal map in Methods, S1 Fig). An example of rhodopsin-immunoreactive cells (arrows) taken at superior-temporal region at each stage was shown next to the retinal map. At each stage, more rhodopsin-immunoreactive cells were apparent in the superior-temporal region compared to other areas of the saline-treated (Fig 2A, 2C and 2E) and TIMP1 treated *rd1* retinas (Fig 2B, 2D and 2F). At P30, more rhodopsin immunoreactive cells were visible in the TIMP1-treated retina (Fig 2B) than that of the saline-treated retina (Fig 2A). In later stages of the saline-treated retina, sparse and scattered rod cells were observed (Fig 2C and 2E). In contrast, more rod cells were found in TIMP1-treated *rd1* retinas compared to the saline-treated retinas in the later stage of retinal degeneration (Fig 2D and 2F).

We examined the mean total number of rod cells in saline-treated and TIMP1-treated *rd1* retinas. The mean total number of rod cells in saline-treated retinas at P30, P35, and P45 were 3,427±97, 297±15, and 113±3, respectively. The mean total number of rod cells in TIMP1-treated retinas showed higher numbers of 7,312±252, 1,934±46, and 474±33, respectively. The two-way ANOVA analysis showed significant differences between the mean of saline-treated and TIMP1-treated retinas at P30, P35 (****P<0.0001) and P45 (*P<0.05), Fig 3A). For the control, we measured the areas of the retinas used for counting rod cells to verify that no sampling errors occurred between the saline-treated and the TIMP1-treated retinas (Fig 3B). The mean retinal areas of saline-treated *rd1* retinas at P30, P35, and P45 were 10.2±0.3 mm^2^, 10.1±0.2 mm^2^, and 10.2±0.2 mm^2^, respectively. The mean retinal areas of TIMP1-treated *rd1* retinas at P30, P35, and P45 were 10.4±0.3 mm^2^, 10.3±0.3 mm^2^, and 10.4±0.1 mm^2^, respectively (Fig 3B). The two-way ANOVA analysis showed no significant differences between the means of the two groups of retinas and the different postnatal days (P = 0.60 at P30, P = 0.58 at P35, P = 0.65 at P45). These results clearly indicated that TIMP1 treatment slows rod cell death at P30, P35, and P45 *rd1* mouse retinas.

### Scotopic electroretinograpy

**Fig 4:**
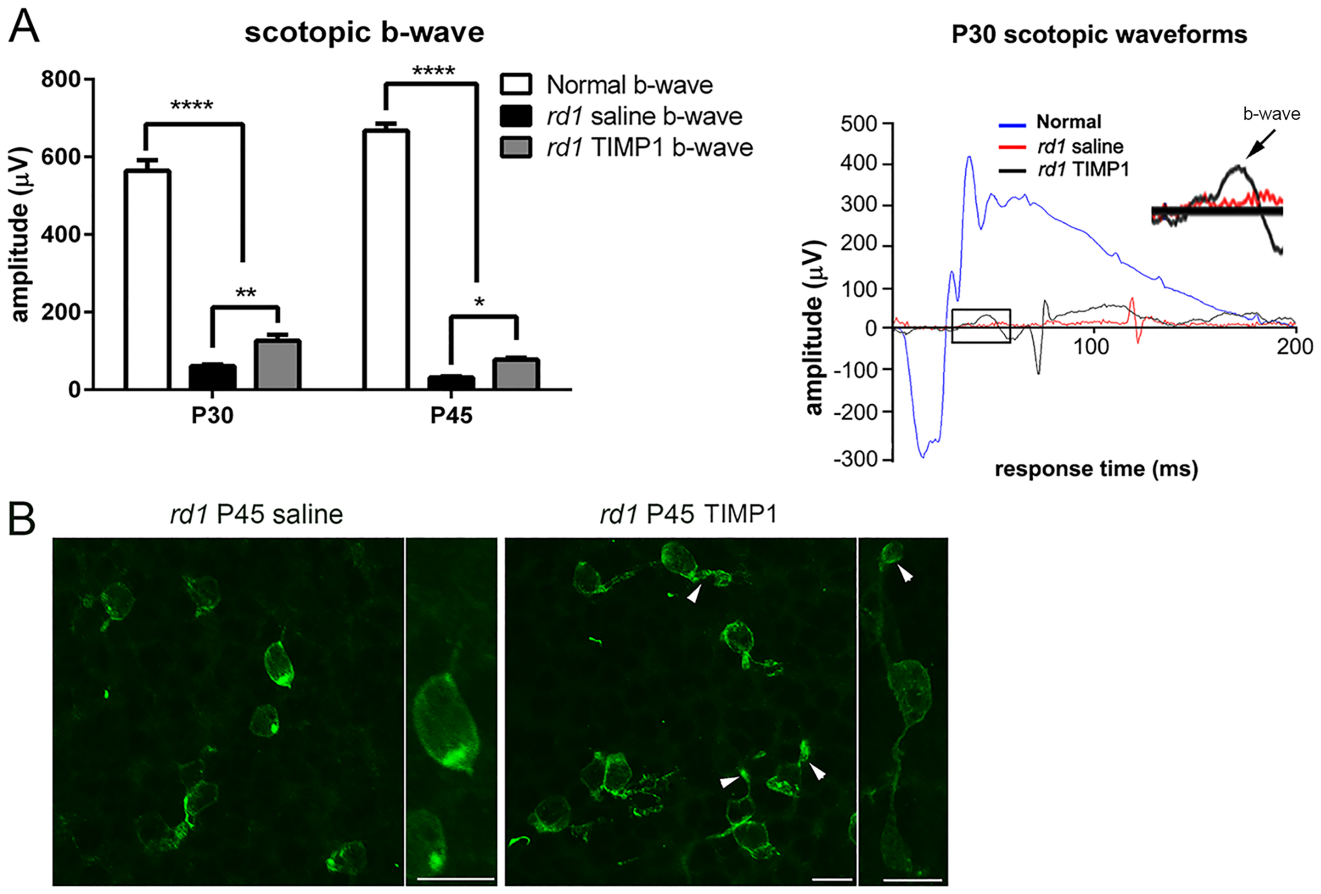
Scotopic ERG recordings from saline-treated and TIMP1-treated *rd1* retinas. Representative b-wave (A) from saline-treated and TIMP1-treated retinas is shown in Fig 4. In P30 and P45, the b-wave amplitudes of TIMP1-treated *rd1* retinas were significantly higher than those of saline-treated *rd1* retinas but not to the degree of normal retina (**** P<0.0001, ** P = 0.004, * P = 0.03). Representative waveforms of the scotopic ERG response from each group were generated. Rhodopsin immunoreactivity was in the cell bodies and some processes of rods in saline-treated *rd1* retinas. In contrast, rhodopsin immunoreactivity was in some segments (arrowheads), cell bodies, and processes of rods in TIMP1-treated *rd1* retinas (C, S2 Fig). The inset shows higher magnification of b-wave (arrow) of both saline-treated and TIMP1-treated P30 retinas. Data represents mean ± SEM, **** P<0.0001, ** P = 0.004, * P = 0.03; P, postnatal. Scale bar = 10 μm.

The effect of TIMP1 treatment on retinal function was measured using ERG. The scotopic ERG was recorded and the amplitudes of the b-wave were analyzed (Fig 4A). In addition, an example of waveforms of the scotopic ERG responses from P30 normal (blue), P30 saline-treated (red), and P30 TIMP1–treated (black) retinas were generated. The amplitudes for the resulting b-wave responses at the light flash intensity of 3000 mcd.s/m^2^ (i.e., optimal light stimulus for doing ERG recordings in research animals and humans [46]) was plotted in this study. The scotopic a-wave was highly variable and barely detectable in both saline-treated, and TIMP1 treated retinas compared to normal retinas. The amplitudes of the b-waves (Fig 4A) in *rd1* saline-treated retinas were considerably weaker. The mean b-wave amplitudes of saline-treated *rd1* retinas at P30 and P45 were 61.2±3.6 μV and 31.3±3.2 μV, respectively. In contrast, the mean b-wave amplitudes of TIMP1-treated *rd1* retinas at P30 and P45 were 126.6±14.9 μV and 77.9±4.5 μV, respectively. Thus, b-wave amplitudes of TIMP1-treated *rd1* retinas were significantly higher than those of saline-treated *rd1* retinas (** P = 0.004, * P = 0.03 two-way ANOVA analysis). However, b-wave amplitudes of TIMP1–treated retinas did not reach the degree of amplitude seen in normal retinas (P30, 564.6±26.8 μV; P45, 667.9±17.8 μV, **** P<0.0001, two-way ANOVA analysis).

In Fig 4B, we illustrate an example of whole-mount retinas (i.e. collected after ERG recordings) processed for rhodopsin immunohistochemical staining at P45 taken at the central part of the superior-temporal region (i.e. where rod protection is observed) of *rd1* saline and *rd1* TIMP1 retinas. In *rd1* saline-treated retina, rhodopsin immunoreactivity was in the cell bodies and some processes of rods. In *rd1* TIMP1-treated retinas, immunoreactive cell bodies, processes, and some outer segments (arrowheads) were visible with anti-rhodopsin antibody. Thus, TIMP1 partially delayed the deterioration of outer segments of rods. Higher magnification of panel showed an example of a rod that stained with rhodopsin antibody in the saline-treated and TIMP1-treated retinas.

### TIMP1 plays a protective role via ERK 1/2 activation in *rd1* retina

**Fig 5:**
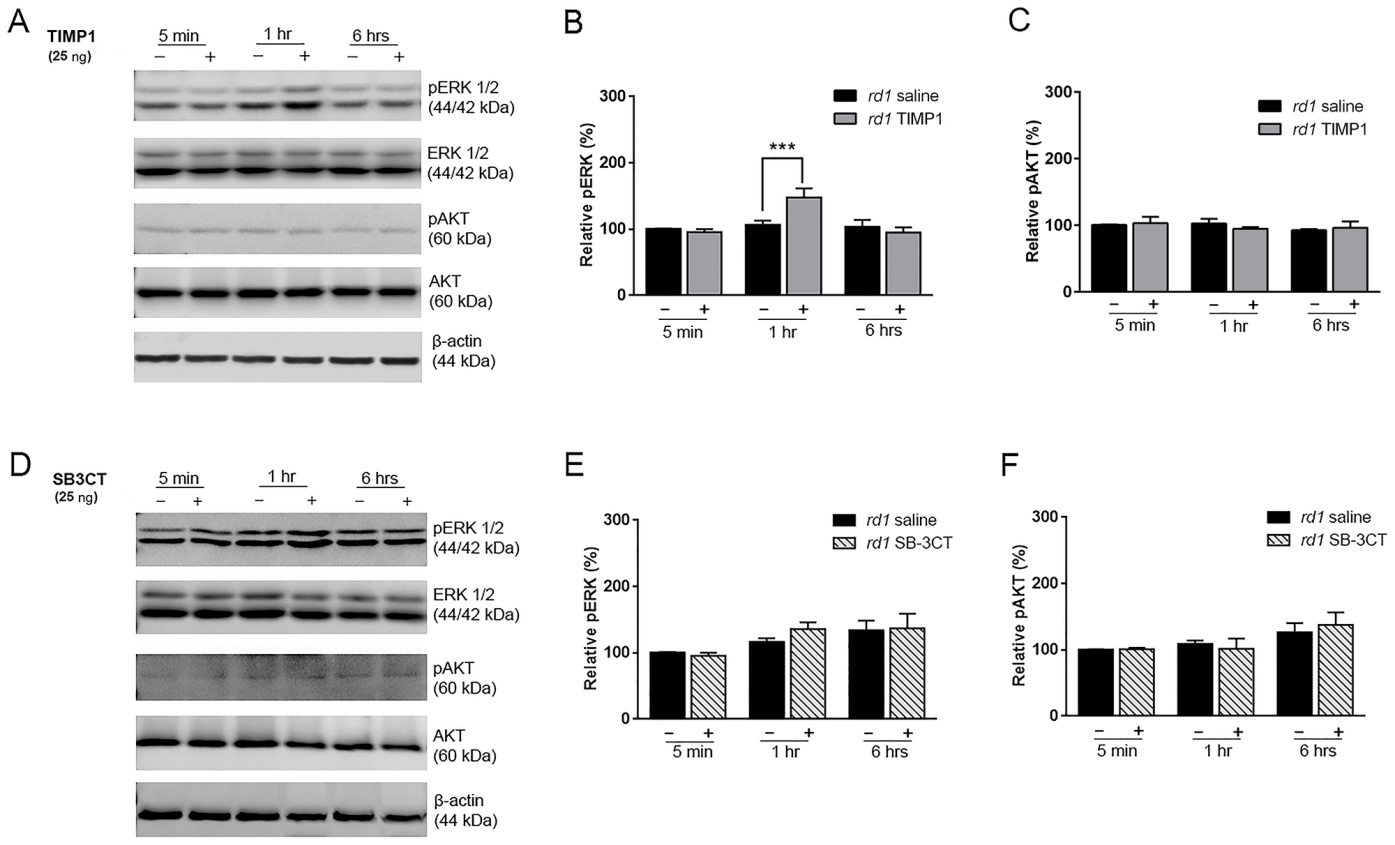
Induction of pERK1/2 by TIMP1 in *rd1* mouse retina. Immunoblot analysis of SDS-PAGE transferred samples of phosphorylated ERK1/2 (44/42 kDa) and AKT (60 kDa) were examined from saline- (-) and TIMP1-treated (+) (A-C) and SB-3CT-treated (D-F) *rd1* retinas. Retinas were collected at 5 min, 1 hour, and 6 hours after injection at P15 and processed. Activation of ERK1/2 was detected at 1 hour after TIMP1 injection (A, B). No pAKT increase was detectable in TIMP1 treated retinas (A, C). In addition, no detectable increase in either pERK1/2 or pAKT was noted in SB-3CT-treated retinas (D, E, F). Densitometry analysis of immunoblots was generated in the histogram. Immunoreactive β-actin served as the loading control to gain relative pERK1/2 and pAKT activation value. Data represent mean ± SEM, *** P<0.001. ERK, Extracellular Signal-regulated Kinase-1; pERK, phosphorylated Extracellular Signal-regulated Kinase-1; AKT, Protein kinase B; pAKT, Phosphorylated Protein kinase B.

Our data demonstrate that SB-3CT, a specific MMP2 and MMP9 inhibitor, does not impact or extend rod survival in *rd1* retinas (Fig 1). However, our results highlight and illustrate TIMP1 plays a significant neuroprotective role through a yet unknown survival signaling pathway rather than through MMP inhibition [29, 30]. TIMP1 was shown to play a role in cell survival independently of its inhibitory function to MMPs via specific cellular signaling pathways such as the ERK and AKT pathways in various cell types (e.g., breast epithelial cells and lung cancer cells) [28]. To explore these alternative independent pathways, we injected saline, SB-3CT or TIMP1 to P15 *rd1* eyes and performed immunoblot analysis on the retinas. The retinas were collected at 5 minutes, 1 hour, and 6 hours. ERK 1/2 phosphorylation (pERK 1/2) demonstrated a significant increase at 1 hour after TIMP1 treatment (Fig 5A). In contrast, pERK 1/2 expression did not change among the saline-treated retinas. However, no significant difference in expression of pAKT between TIMP1 treated and saline-treated groups was detected (Fig 5A). Densitometry of pERK-immunoreactive proteins was performed (Fig 5B). After TIMP1 treatment, the pERK 1/2 expression was significantly higher at 1 hour compared to that of saline-treated retinas (***P<0.001). In contrast, there were no significant differences in the expression levels of pAKT between saline- and TIMP1-treated retinas (Fig 5C). We also injected SB-3CT to examine if inhibition of MMP9 and MMP2 affect AKT or ERK pathways. We did not observe changes of pERK 1/2 or pAKT levels between saline-treated and SB-3CT-treated groups (Fig 5D, 5E and 5F, S3 Fig). Our results clearly demonstrate that TIMP1 plays a protective role through ERK 1/2 activation rather than AKT activation in *rd1* retina.

### TIMP1 protects rod photoreceptor cells survival through activation of BAX suppression

**Fig 6:**
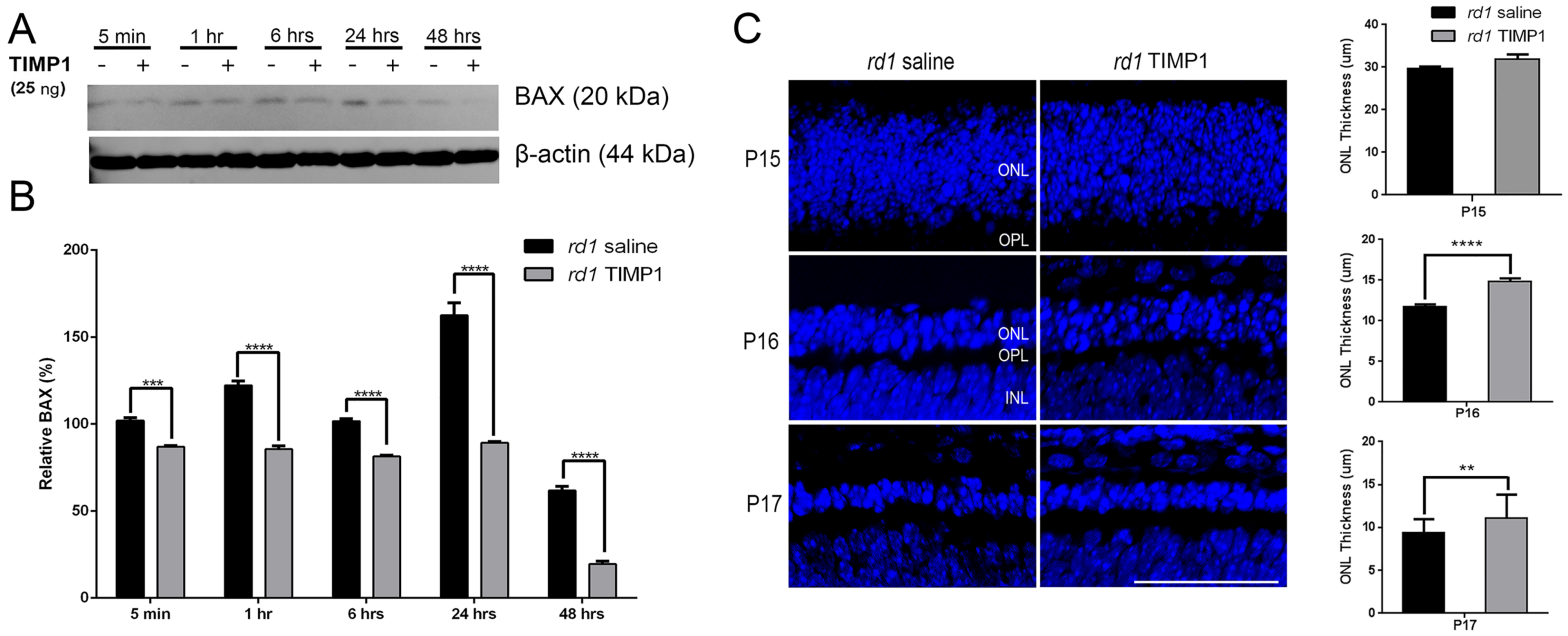
Suppression of BAX expression by TIMP1 in *rd1* retina. Retinas were collected at 5 minutes, 1 hour, 6 hours, 24 hours, and 48 hours after injection at P15. BAX expression was significantly decreased from 5 minutes after TIMP1 injection (+) compared to saline groups (-). Densitometry analysis of BAX expression was shown by measuring the intensity relative to the control β-actin (B). Data represents mean ± SEM, ****P<0.0001, ***P<0.005. Confocal micrographs were taken from vertical cryostat sections (10 μm-thick) processed for TOPRO-3 staining (blue) in the P15 (i.e. 1 hour after TIMP1 post-injection), P16, and P17 saline-treated and TIMP1-treated retinas. Histogram shows the thickness of ONL in the different comparisons. Data represents mean ± SEM,**** P<0.0001, **P<0.005; scale bar = 50 μm.

Several studies have reported that TIMP1 played a role in anti-apoptosis by suppressing BAX in cancer cells [14] and mouse bone marrow stromal cell line [53]. In RP rodent models (e.g. *rd1*, Rhodopsin knockout, and rhodopsin P23H mice), upregulation of BAX was suggested to be linked to apoptosis [54]. In the current study, we examined BAX (20 kDa) expression in the retina after TIMP1 treatment at P15 by immunoblot analysis. A significant decrease in BAX expression was detected from 5 minutes to 48 hours after TIMP1 treatment, compared to saline treatment groups (Fig 6A). Quantitative analysis was performed by measuring the immunoreactive BAX band intensity relative to control (Fig 5B). The BAX expression pattern was significantly lower from 5 minutes to 48 hours than that of saline-treated retinas (****P<0.0001, ***P<0.01, two-way ANOVA analysis). Immunoreactive β-actin was shown as a loading control to obtain relative BAX expression. We also examined the impact of TIMP1 treatment on the outer nuclear layer (ONL) thickness in the different comparisons (i.e. *rd1* saline vs. *rd1* TIMP1, Fig 6C). A previous report showed that the entire phagocytosis process of a single rod, from initial microglial contact to eventual intracellular breakdown, occurs over the time-scale of ≈1 hour [55]. Therefore, we examined the impact of TIMP1 on ONL starting from P15. At P15 (i.e. 1 hour after saline or TIMP1 treatment) the appearance of the ONL of *rd1* saline (29.6 ± 0.6 μm) was similar to *rd1* TIMP1 (31.9 ± 1.0 μm). However, intravitreal injection of TIMP1 at P15 resulted in significant protection of ONL when examined at P16 (24 hours) and P17 (48 hours) after TIMP1 treatment compared to saline-treated groups (Fig 6C). Measurement of the ONL thickness showed that the thickness in the *rd1* saline retinas (P16, 11.7 ± 0.3 μm; P17, 9.3 ± 0.2 μm) was significantly thinner than in *rd1* TIMP1-treated retinas (P16, 14.8 ± 0.3 μm; P17, 11 ± 0.5 μm, **** P<0.0001, **P<0.005). These results indicated that TIMP1 played a significant role [56] in rod survival in *rd1* retina through BAX suppression.

### Does TIMP1 protect cone photoreceptor cell survival?

**Fig 7:**
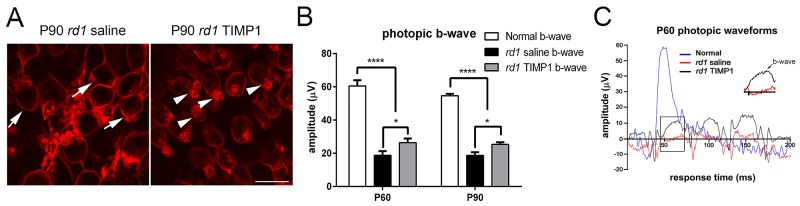
Photopic ERG responses in saline-treated and TIMP1-treated *rd1* retinas. Confocal micrographs of whole-mounts immunohistological stained for M-opsin in P90 saline-treated and P90 TIMP1-treated retinas (A). In the TIMP1-treated retina, M-opsin immunoreactivity was visible in the cell membrane and outer segments that are shortened and distorted (arrowheads). Photopic ERG recordings from saline-treated *rd1* and TIMP1-treated *rd1* retinas were shown (B). In addition, an example of waveforms of the photopic ERG responses from P60 normal, P60 saline-treated, and P60 TIMP1–treated retinas were generated (C). The inset shows higher magnification of b-wave (arrow) of both saline-treated and TIMP1-treated P60 retinas. Data represents mean ± SEM; **** P<0.0001, *P<0.05; scale bar = 10 μm; P, postnatal.

Previously, we observed that exogenous application of TIMP1 significantly protected the outer segments of the cones in the retinas of S334ter rats [34]. Since rod photoreceptor cell survival was positively affected by TIMP1 (Figs 1–4), we wanted to see if it also affected the cone survival in the *rd1* mouse retina. Intravitreal injection of TIMP1 was done at P45, after the majority rod photoreceptor cells were degenerated (Figs 2 and 3). In Fig 7A, we showed an example of a whole-mount retinas processed for M-opsin immunohistochemical staining at P90 taken at the central part of the superior-temporal region (i.e. where rod protection was observed in earlier stage of RP) of *rd1* saline-treated and *rd1* TIMP1-treated retinas. In P90 whole-mount retinas, M-opsin cones were devoid of outer segments in *rd1* saline-treated retinas. M-opsin immunoreactivity was mainly present in the cell membrane (arrows). Whereas in TIMP1 treated retina, M-opsin immunoreactivity was in the cell membrane and outer segments that are shortened and distorted (arrowheads). The shortening or loss of cone outer segments and mislocalization of opsins in *rd1* were consistent with previous studies [34, 57, 58]. Our result showed that TIMP1 treatment prolongs some portion of outer segments of cones at advanced stages of retinal degeneration.

The physiological effect of TIMP1 on cone photoreceptor visual function was measured using photopic ERG (Fig 7B). In addition, an example of waveforms of the photopic ERG responses from P60 normal, P60 saline-treated, and P60 TIMP1–treated retinas was generated (Fig 7C). The photopic a-wave was highly variable in both saline-treated, and TIMP1 treated retinas compared to normal retinas. Thus, we only show amplitudes of photopic b-wave. The photopic b-wave in saline-treated *rd1* and TIMP1-treated *rd1* retinas were analyzed (Fig 7B). The amplitudes of the resulting b-wave responses at the light flash intensity of 3000 mcd.s/m^2^ were generated in a histogram (Fig 7B). The mean b-wave amplitudes of saline-treated *rd1* retinas at P60 and P90 were 18.8±2.5 μV and 18.7±1.9 μV, respectively. In contrast, the mean b-wave amplitudes of TIMP1-treated *rd1* retinas at P60 and P90 were 26.4±2.5 μV and 25.4±1.2 μV, respectively. Thus, b-wave amplitudes of TIMP1-treated *rd1* retinas were significantly higher than those of saline-treated *rd1* retinas (*P<0.05, two-way ANOVA, Fig 7E and 7F). However, b-wave amplitudes of TIMP1–treated retinas did not reach the degree of amplitude seen in normal retinas (P60, 60.52±3.4 μV; P90, 54.6±1.17 μV, **** P<0.0001, two-way ANOVA analysis).

## Discussion

### Rod survival in superior-temporal region of the *rd1* retina

In this study, our goal was to preserve rod photoreceptors with TIMP1 treatment, which was effective on cone photoreceptor survival in our previous study in S334ter rat retinas [34]. To determine the protective effects of TIMP1 on rods, we performed intravitreal injections on *rd1* mice with TIMP1 at P15 and analyzed retinas at P30, P35, and P45. In addition to finding that TIMP1 increased rod photoreceptor cell number (Fig 3) and more intact rod outer segments through P45 (Fig 4), we also observed more rhodopsin-immunoreactive cells in the superior-temporal region compared to other areas of the saline-treated (Fig 2A, 2C and 2E) and TIMP1 treated *rd1* retinas (Fig 2B, 2D and 2F).

In *rd1* retina, the degeneration of rods start around P8-10 [59, 60], shows a dramatic peak between P12-15 [36, 61, 62], and is almost gone by P36 [63]. Similar to other models of photoreceptor degeneration, the *rd1* model studied here shows a clear center to periphery gradient (Fig 2) of rod cell death [64–66]. Almost completion of rod death was observed around P35 with a few far peripheral rods that persisted around P45 (Fig 2C and 2E) [63]. The cause of this gradient has been hypothesized to be connected to differences in transcription factor expression levels between the center and periphery cells [67]. It could also be the result of a positive feedback loop, where rods undergoing apoptosis may induce neighboring rods to undergo apoptosis either by modifying the extracellular matrix and/or reducing trophic factors [68, 69]. Thus, more rod cell death in a certain region of the retina leads to a more toxic environment for neighboring rods [70–72]. In saline-treated and TIMP1-treated retinas, rods in the superior region remain viable longer as indicated by the higher rod cell density (Fig 2). Our result may be due to the existence of more rod survival in this region as previously described in normal mouse retina [73, 74]. However, administration of TIMP1 showed more survival of rods in the superior region of the retinas compared to saline-treated retinas (Fig 2). The delay of rod death in TIMP1 treated *rd1* retina suggests that TIMP1 is more effective for cellular viability in the denser region of cells, affecting cell to cell, cell to extracellular matrix (ECM) interaction, and protecting extracellular matrix (ECM)-bound growth factors [6]. During retinal degeneration, ECM structural and functional properties are changed. The ECM change leads to alterations in the expression levels of matrix metalloproteinase (MMP)/TIMP1 [68, 69, 75, 76]. For example, TIMP-3 mRNA was significantly up-regulated in human RP and Sorsby’s fundus dystrophy conditions [77, 78]. Thus, a hypothesis to explain our results is that exogenous application of TIMP1 compensated the altered enzymatic balance and the properties of the ECM (e.g., integrins and various cytoplasmic proteins [2, 79]) to protect rod photoreceptors and other supporting cells [37] in that region.

### TIMP1 treatment influences rod survival in an MMP-independent manner

Unlike the S334ter rat transgenic retina model, in which SB-3CT preserved rod cell viability by suppressing up-regulated MMP9 activity, SB-3CT did not preserve rod cells in the *rd1* mouse RP model. The rod cell densities in saline-treated and SB-3CT treated retinas showed similar results (Fig 1A). In contrast, TIMP1, an inhibitor of MMPs (specifically MMP9) [17], treated retinas showed increased rod cell densities. Thus, our results suggest that TIMP1 influenced rod survival in an *MMP-independent manner* [19]. Further support for this observation comes from the zymography results in *rd1* retinas (Fig 1B). Gelatinolytic activity of MMP9 showed no significant difference in both normal and *rd1* retinas at P10, P14, and P18 (Fig 1B and 1C). Gelatinolytic activity of MMP2 was only shown in *rd1* postnatal retinas (Fig 1B). In our previous rat retina study, we showed the gelatinolytic activity of the MMP9 was elevated, while MMP2 was relatively unchanged in S334ter retinas. With treatment of SB-3CT, the gelatinolytic activity of both MMPs was suppressed and subsequently delayed the rod cell death [38]. In contrast, our results suggest that rod cell death in *rd1* mouse retina is not highly associated with the level of MMP9 and MMP2 [38], which is supported and documented in the literature by alternative biological functions of TIMP1 [8, 80, 81]. TIMP1 treatment definitely mediates anti-apoptotic activity through ligand-receptor interactions via a FAK/PI3K/AKT pathway and ERK in central nervous system (CNS) injury, inflammation, and cancer cells [33, 82, 83]. In *rd1* mouse retina, we observed up-regulation of ERK by immunoblot analysis (Fig 5). Currently, we do not know if ERK expression is up-regulated in photoreceptor cells in *rd1* mouse retinas; however, ERK is expressed in Müller cells and ERK pathways are present in the photoreceptor cells with basal levels of phosphorylation with recombinant cysteine-rich heparin-binding protein 61 [84]. It is well known that glial cells are regulated by signals from the ECM [85]. In addition, there is evidence that TIMP1 can affect the glial cells by modulating focal adhesion of integrin receptors and various cytoplasmic proteins in the ECM. Furthermore, Müller cells play a role in the regulation of expression and production of TIMP and MMP through a feedback system via the ECM [37, 86, 87]. Therefore, we postulate that TIMP1 stimulates ERK pathways in the Müller cells through ECM to release of cytokines, neurotrophic and growth factors that affect and preserve the photoreceptor cells leading to prolonged survival [84, 86, 88–97].

### TIMP1 treatment delays rod photoreceptor cell death in *rd1* retina

In *rd1* retina, BAX is expressed in the early stage of retinal development [98]. BAX plays a central role in apoptosis. Normally, BAX is in the cytosol of healthy cells, but with activation in response to apoptotic signals, it is rapidly translocated into the mitochondrial outer membrane and releases cytochrome c [99]. The mitochondrial function is essential to maintain photoreceptors against retinal degeneration [100–102]. Thus, one of the critical events inhibiting the apoptosis commitment step is suppressing BAX activity. Therefore, increased expression of BAX in *rd1* retina and its down-regulation by TIMP1 treatment suggests that TIMP1, in part, protect rod photoreceptors by suppressing the important mitochondrial pathway leading to the onset of cell death. This hypothesis is supported by published data that TIMP1 is involved in inhibiting apoptosis by suppressing BAX in cancer cells, bone marrow stromal cell line, and mesangial cells [14, 53, 103]. Our results also demonstrate that TIMP1 treatment slows the attenuation of ONL thickness at P16 and P17 (Fig 6C). This finding suggests that down-regulation of BAX from 5 minutes after TIMP1 post-injection (Fig 6B) leads to a reduction of cell death. Thus, our results clearly demonstrate that inhibition of BAX after 5 minutes of TIMP1 post-injection is significant enough to cause survival effects on the number of rods in the *rd1* retinas at early stages (Figs 2 and 3).

Does activation of ERK by TIMP1 suppress BAX? We observed up-regulation of pERK after 1 hour TIMP1 post-injection. Currently, we cannot rule out the possibility that the pERK may be involved in the suppression of BAX after 1 hour of TIMP1 post-injection. However, there is evidence that activation of ERK may, in part, lead to the regulation of the BAX expression after TIMP1 treatment [33, 82, 83].

### Effects of TIMP1 treatment in later stages of retinal degeneration in *rd1* mouse retina

Previous papers showed that cones survive for several months after degeneration of the rods [63, 104–106]. There are several reasons that lead cones to subsequently die. These include oxidative stress [107] from rod degeneration [108, 109], toxic substrates released by dying rods and loss of trophic factors provided by rods [71, 72, 110], reducing the flow of nutrients from retinal pigment epithelium to cones [111]. However, a recent study shows that rod-derived cone viability factor stimulates the glucose transporter and thereby increases the glucose entry into cones to prevent secondary cone death in RP retina [112]. In *rd1* retina, cone degeneration starts before P12 [57], but the relatively constant number of cones remain until P65, then proceed to degeneration within six postnatal months [57, 106, 113]. Previous papers showed that there are significant changes in number of both M-opsin and S-opsin cones in retinal degeneration mice from P60 to P90 [57, 104, 106]. With TIMP1 treatment, we found that rods and cones maintained their outer segments at the P45 and P90, respectively (Figs 4 and 7, arrowheads). In addition, we observed The significant differences of scotopic b-wave or photopic b-wave amplitudes are observed between saline-treated and TIMP1-treated retinas. Our b-wave ERG responses also suggest that the existence of healthier outer segments of rods and cones in TIMP1-treated retinas. Without outer segments, rods and cones lose their photon-sensing function. In *rd1* retinas, entire rhodopsin and M-opsin immunoreactive rods and cones are labeled, respectively. The mislocalization of rhodopsin and cone opsins in *rd1* was consistent with previous studies [58, 114–118]. The redistributed rhodopsin and cone opsins may still hold photon perception mechanisms, but their efficiency of signal transference to post-synaptic neurons may be affected, leading to lower ERG responses. At P30 saline-treated retina, abnormal rod ERG function was detected, which confirms an early onset of retinal degeneration in this mouse model. Typically, the amplitude of b-wave of scotopic ERG is ~650 μV at the similar light intensity. The amplitude of b-wave of photopic ERG in mouse retina at similar light intensity is ~15 μV and ~60 μV [119, 120]. Thus, lower amplitude of scotopic b-wave (Fig 4) and photopic (Fig 7) b-wave in saline-treated retinas indicates the changes in the efficiency of the synaptic connection of photoreceptors to bipolar cells [34, 64, 121–123]. Furthermore, dysfunction and death of photoreceptors cause secondary structural change and remodeling in post-synaptic neurons. These changes include the retraction of bipolar cell dendrites and altered glutamate receptors expression [38, 58, 64, 121–126]. This is supported by detailed studies that showed aberrant functional ionotropic receptors in the ON bipolar cells in the *rd1* retina, leading to a reduction in b-wave amplitude [64, 127]. Taken together, TIMP1 treatment slowed the attenuation of ONL and protected some outer segments of photoreceptors. However, the amplitude of b-wave appeared to be limited to the compensation by TIMP1 in early and later stages of *rd1*, because the maximum of scotopic b-wave or photopic b-wave amplitudes in TIMP1 treated retinas were considerably lower than normal (Figs 4 and 7). In the future, we will analyze potential survival effects of TIMP1 in long-term by developing TIMP1-secreting microdevices and implanting them intravitreally into the eyes of animals with RP. We believe that sustained delivery of TIMP1 may preserve photoreceptors and maintain their function to preserve vision.

## Conclusion

We have shown the neuroprotective potential of TIMP1 treatment in *rd1* retina. Although TIMP1 is well documented as an anti-apoptotic mediator, it is still unclear how TIMP1 regulates cell survival in neurons including the retina. Our findings provide evidence that TIMP1 significantly reduced BAX activity and delayed attenuation of the outer nuclear layer; in addition to inducing phosphorylated ERK 1/2 signaling pathway in *rd1* retina.

## Supporting information

S1 Fig Composite image of P30 *rd1* saline-treated whole-mount retina. Rhodopsin immunoreactivity was shown throughout the P30 *rd1* saline-treated whole-mount retina. The white line indicates the border of the retina. Scale bar = 1mm, insect, 50 μm.
(DOCX)

S2 Fig Confocal micrographs of vertical sections double-labeled with M-opsin (red) and rhodopsin (green) in saline-treated and TIMP1 treated *rd1* retinas at P30. Rhodopsin immunoreactivity and M-opsin immunoreactivity in vertical section of saline-treated and TIMP1-treated P30 retinas. Scale bar = 50 μm.
(DOCX)

S3 Fig Immunoblot analysis of pERK1/2 in the SB-3CT-treated *rd1* retina. Expression of pERK 1/2 at 60 minutes and 90 minutes between saline-treated and SB-3CT-treated groups showed no difference. β-actin was used as a loading control to obtain relative pERK1/2 expression.
(DOCX)

S4 Fig Thickness of INL in saline-treated and TIMP1-treated rd1 retinas. Histograms show the thickness of INL in the different comparisons. Data represents mean ± SEM. The data showed no significant differences in thickness of INL in P16 (A, P = 0.19) and P17 (B, P = 0.84).
(DOCX)

S1 Table Quantification of rods in saline-treated, TIMP1-treated, and SB-3CT-treated *rd1* retinas. The rhodopsin-immunoreactive rods were measured from the 0.25x0.25 mm^2^ sampling areas (for details, see Methods) of saline-treated, TIMP1-treated, and SB-3CT-treated *rd1* retinas (Fig 1).
(DOCX)

S2 Table Quantification of rhodopsin-immunoreactive cells in saline-treated and TIMP1-treated *rd1* whole-mount retinas. The total number of rods was measured from the whole-mount retinas of saline-treated and TIMP1-treated *rd1* retinas (Fig 2).
(DOCX)

S3 Table The retinal area of saline-treated and TIMP1-treated whole-mount *rd1* retinas. The retinal area of whole-mounts was measured from the P30, P35, and P45 saline-treated and TIMP1-treated *rd1* retinas (Fig 3B).
(DOCX)

S4 Table The amplitudes of b-wave scotopic ERGs in normal, saline-treated and TIMP1-treated retinas. Amplitudes of b-waves were measured from P30, and P45 normal, saline-treated and TIMP1 treated *rd1* retinas (Fig 4A).
(DOCX)

S5 Table Quantification of pERK and pAKT expression in saline-treated vs. TIMP1-treated retina by immunoblot analysis. Immunoblot analysis shows up regulation of pERK1/2 and not pAKT in the TIMP1 treated *rd1* retina, compared to saline-treated *rd1* retina. β-actin was used as a loading control to obtain relative pERK1/2 and pAKT expression (Fig 5A–5C).
(DOCX)

S6 Table Quantification of pERK and pAKT expression in saline-treated vs. SB-3CT-treated retina by immunoblot analysis. Immunoblot analysis shows no detectable changes in expression of pERK1/2 and pAKT in the SB-3CT treated *rd1* retina, compared to saline-treated *rd1* retina. β-actin was used as a loading control to obtain relative pERK1/2 and pAKT expression (Fig 5D–5F).
(DOCX)

S7 Table Quantification of BAX expression in saline-treated vs. TIMP1-treated retinas by immunoblot analysis. Immunoblot analysis shows suppression of BAX from 5minutes to 48 hours after TIMP1 injection at P15. β-actin was used as a loading control to obtain relative BAX expression (Fig 6).
(DOCX)

S8 Table The amplitudes of b-wave photopic ERGs in normal, saline-treated and TIMP1-treated retinas. Amplitudes of b-wave were measured from P60 and P90 normal, saline-treated and TIMP1 treated *rd1* retinas (Fig 7).
(DOCX)
