# Supplementary figures and images for: Tissue inhibitor of metalloproteinases 1 enhances rod survival in the rd1 mouse retina
Source: PLoS One. 2018 May 9;13(5):e0197322. doi: 10.1371/journal.pone.0197322 (PMC5942829; doi:10.1371/journal.pone.0197322)

**S1. Fig. Composite image of P30 *rd1* saline-treated whole-mount retina.**


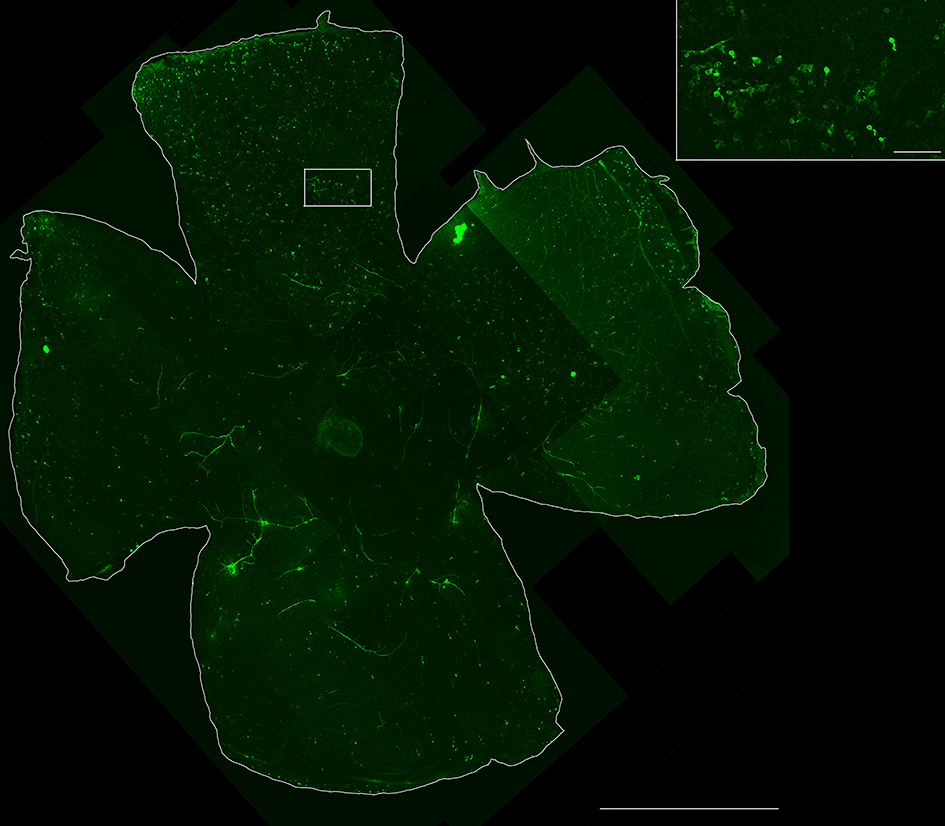

Supplement: S1 Fig — Rhodopsin immunoreactivity was shown throughout the P30 rd1 saline-treated whole-mount retina. The white line indicates the border of the retina. Scale bar = 1mm, insect, 50 μm. (DOCX) [file pone.0197322.s001.docx]

**S3 Fig. Immunoblot analysis of phosphorylated ERK1/2 in saline-treated and SB-3CT-treated *rd1* retinas.**


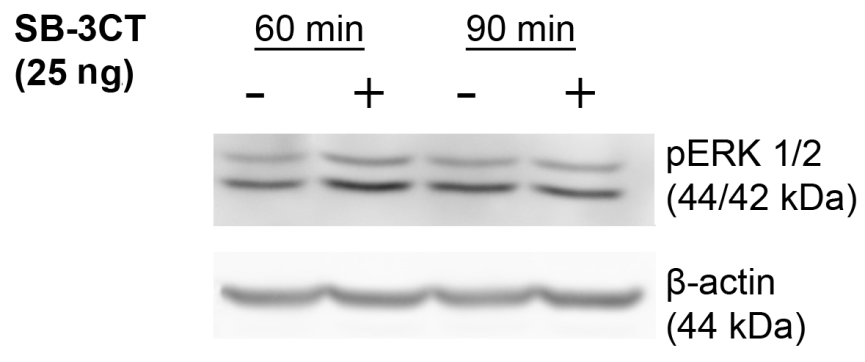

Supplement: S3 Fig — Expression of pERK 1/2 at 60 minutes and 90 minutes between saline-treated and SB-3CT-treated groups showed no difference. β-actin was used as a loading control to obtain relative pERK1/2 expression. (DOCX) [file pone.0197322.s003.docx]

**S4 Fig. Thickness of INL in saline-treated and TIMP1-treated *rd1* retinas.**


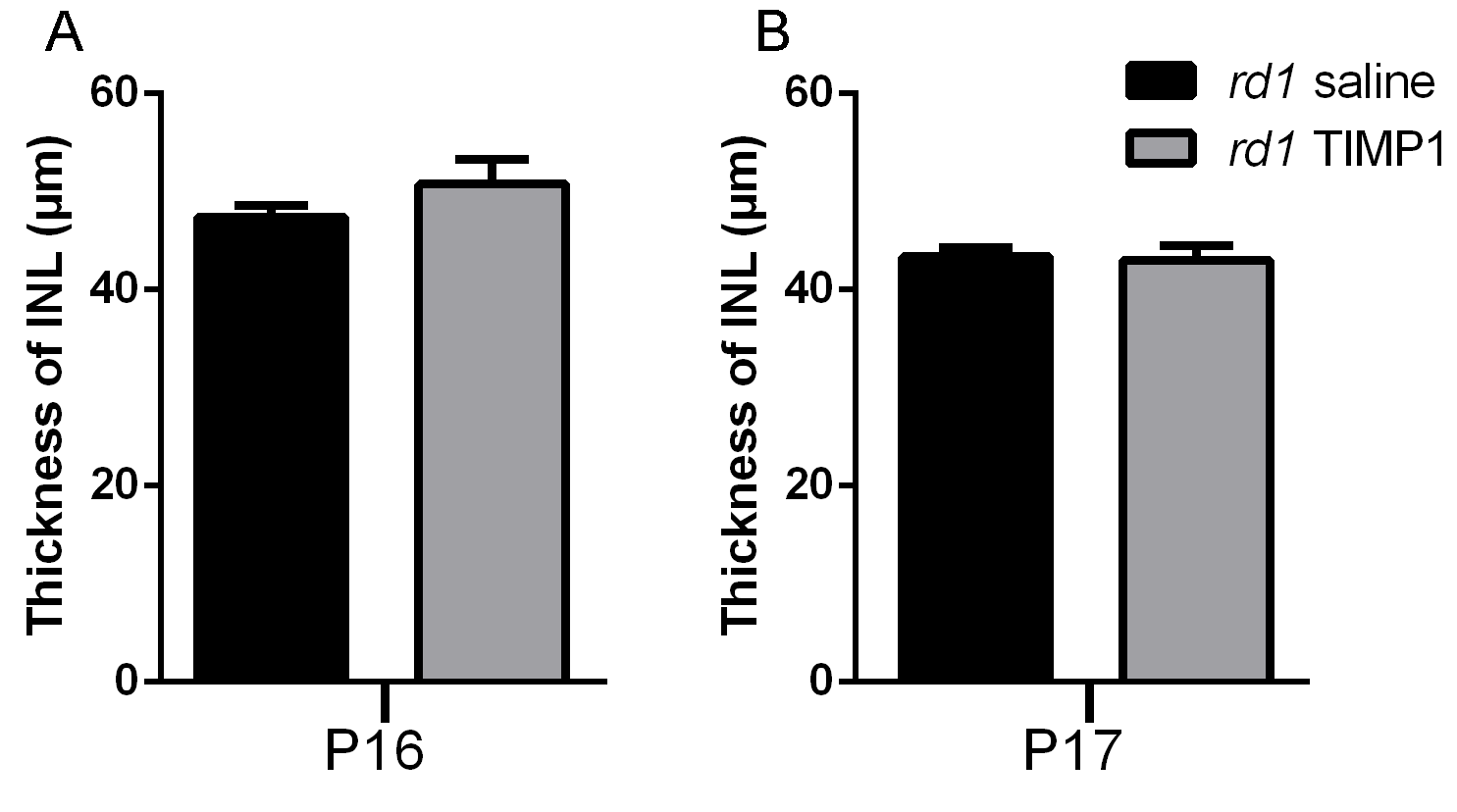

Supplement: S4 Fig — Histograms show the thickness of INL in the different comparisons. Data represents mean ± SEM. The data showed no significant differences in thickness of INL in P16 (A, P = 0.19) and P17 (B, P = 0.84). (DOCX) [file pone.0197322.s004.docx]
