# Supplementary material for: Tissue inhibitor of metalloproteinases 1 enhances rod survival in the rd1 mouse retina
Source: PLoS One. 2018 May 9;13(5):e0197322. doi: 10.1371/journal.pone.0197322 (PMC5942829; doi:10.1371/journal.pone.0197322)

**S2 Fig. Confocal micrographs of vertical sections double-labeled with M-opsin (red) and rhodopsin (green) in saline-treated and TIMP1 treated *rd1* retinas at P30.**


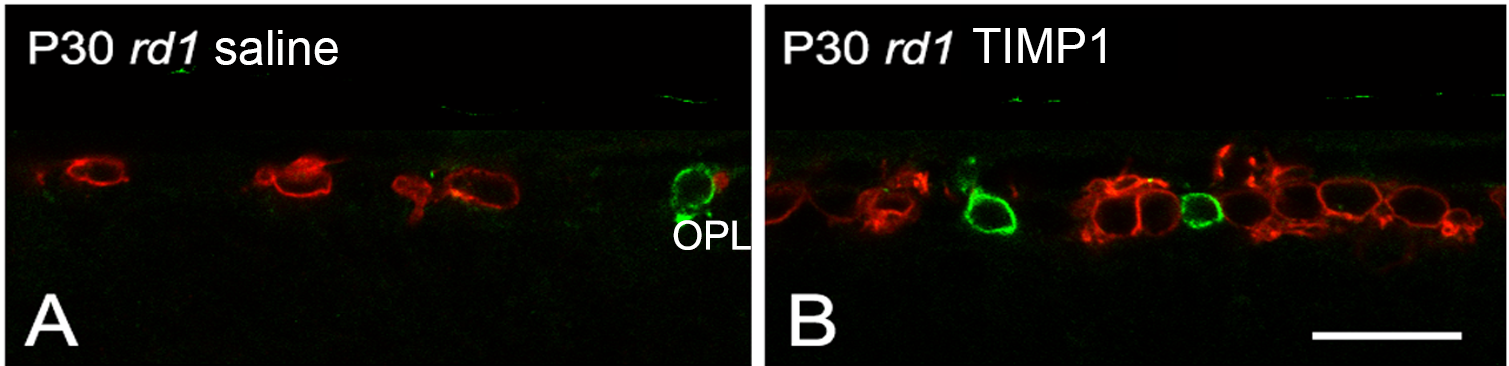

Supplement: S2 Fig — Rhodopsin immunoreactivity and M-opsin immunoreactivity in vertical section of saline-treated and TIMP1-treated P30 retinas. Scale bar = 50 μm. (DOCX) [file pone.0197322.s002.docx]
