# Supplementary material for: Tissue inhibitor of metalloproteinases 1 enhances rod survival in the rd1 mouse retina
Source: PLoS One. 2018 May 9;13(5):e0197322. doi: 10.1371/journal.pone.0197322 (PMC5942829; doi:10.1371/journal.pone.0197322)
Supplement: S1 Table — The rhodopsin-immunoreactive rods were measured from the 0.25x0.25 mm2 sampling areas (for details, see Methods) of saline-treated, TIMP1-treated, and SB-3CT-treated rd1 retinas (Fig 1). (DOCX) [file pone.0197322.s005.docx]

**S1 Table. Quantification of rods in saline-treated, TIMP1-treated, and SB-3CT-treated rd1 retinas.**

|  | | *rd1* saline-treated | | | | *rd1* TIMP1-treated | | | | | *rd1* SB3CT-treated | | | |
| --- | --- | --- | --- | --- | --- | --- | --- | --- | --- | --- | --- | --- | --- | --- |
|  | | Animal 1 | Animal 2 | | Animal 3 | Animal 1 | | Animal 2 | Animal 3 | | Animal 1 | Animal 2 | | Animal 3 |
| P30 | | 414 | 446 | | 426 | 650 | | 736 | 682 | | 439 | 410 | | 452 |
| P35 | | 110 | 136 | | 144 | 326 | | 314 | 352 | | 140 | 132 | | 105 |
| P45 | 24 | | 18 | 26 | | 108 | 118 | | 102 | 22 | | 20 | 30 | |
