# Supplementary material for: Tissue inhibitor of metalloproteinases 1 enhances rod survival in the rd1 mouse retina
Source: PLoS One. 2018 May 9;13(5):e0197322. doi: 10.1371/journal.pone.0197322 (PMC5942829; doi:10.1371/journal.pone.0197322)
Supplement: S2 Table — The total number of rods was measured from the whole-mount retinas of saline-treated and TIMP1-treated rd1 retinas (Fig 2). (DOCX) [file pone.0197322.s006.docx]

**S2 Table. Quantification of rhodopsin-immunoreactive cells in saline-treated and TIMP1-treated *rd1* whole-mount retinas.**

|  | *rd1* saline-treated | | | *rd1* TIMP1-treated | | |
| --- | --- | --- | --- | --- | --- | --- |
|  | Animal 1 | Animal 2 | Animal 3 | Animal 1 | Animal 2 | Animal 3 |
| P30 | 3417 | 3264 | 3601 | 7798 | 6399 | 7184 |
| P35 | 270 | 320 | 301 | 1877 | 2025 | 1990 |
| P45 | 109 | 118 | 111 | 483 | 450 | 490 |
