# Supplementary material for: Tissue inhibitor of metalloproteinases 1 enhances rod survival in the rd1 mouse retina
Source: PLoS One. 2018 May 9;13(5):e0197322. doi: 10.1371/journal.pone.0197322 (PMC5942829; doi:10.1371/journal.pone.0197322)
Supplement: S3 Table — The retinal area of whole-mounts was measured from the P30, P35, and P45 saline-treated and TIMP1-treated rd1 retinas (Fig 3B). (DOCX) [file pone.0197322.s007.docx]

**S3 Table. The retinal area of saline-treated and TIMP1-treated whole-mount *rd1* retinas.**

|  | *rd1* saline-treated | | | *rd1* TIMP1-treated | | |
| --- | --- | --- | --- | --- | --- | --- |
|  | Animal 1 | Animal 2 | Animal 3 | Animal 1 | Animal 2 | Animal 3 |
| P30 | 10.20 | 10.80 | 9.70 | 9.97 | 11.10 | 10.26 |
| P35 | 10.57 | 9.77 | 10.13 | 9.75 | 10.50 | 10.90 |
| P45 | 10.44 | 10.68 | 9.69 | 10.52 | 10.23 | 10.62 |
