# Supplementary material for: Tissue inhibitor of metalloproteinases 1 enhances rod survival in the rd1 mouse retina
Source: PLoS One. 2018 May 9;13(5):e0197322. doi: 10.1371/journal.pone.0197322 (PMC5942829; doi:10.1371/journal.pone.0197322)
Supplement: S4 Table — Amplitudes of b-waves were measured from P30, and P45 normal, saline-treated and TIMP1 treated rd1 retinas (Fig 4A). (DOCX) [file pone.0197322.s008.docx]

**S4 Table. The amplitudes of b-wave scotopic ERGs in normal, saline-treated and TIMP1-treated retinas.**

|  | *rd1* saline-treated | | | | | *rd1* TIMP1-treated | | | | |
| --- | --- | --- | --- | --- | --- | --- | --- | --- | --- | --- |
|  | Animal 1 | Animal 2 | Animal 3 | Animal 4 | Animal 5 | Animal 1 | Animal 2 | Animal 3 | Animal 4 | Animal 5 |
| P30 | 69.8 | 55.4 | 51.3 | 68.3 | 61.2 | 117.3 | 92.0 | 115.5 | 182.0 | 126.0 |
| P45 | 21.7 | 41.0 | 35.0 | 28.0 | 31.0 | 70.0 | 93.7 | 81.0 | 75.8 | 69.0 |

|  | Normal | | | | |
| --- | --- | --- | --- | --- | --- |
|  | Animal 1 | Animal 2 | Animal 3 | Animal 4 | Animal 5 |
| P30 | 547.0 | 623.0 | 517.0 | 633.0 | 503.0 |
| P45 | 698.0 | 720.0 | 628.5 | 634.7 | 658.7 |
