# Supplementary material for: Tissue inhibitor of metalloproteinases 1 enhances rod survival in the rd1 mouse retina
Source: PLoS One. 2018 May 9;13(5):e0197322. doi: 10.1371/journal.pone.0197322 (PMC5942829; doi:10.1371/journal.pone.0197322)
Supplement: S5 Table — Immunoblot analysis shows up regulation of pERK1/2 and not pAKT in the TIMP1 treated rd1 retina, compared to saline-treated rd1 retina. β-actin was used as a loading control to obtain relative pERK1/2 and pAKT expression (Fig 5A–5C). (DOCX) [file pone.0197322.s009.docx]

**S5 Table. Quantification of pERK and pAKT expression in saline-treated vs. TIMP1-treated retina by immunoblot analysis.**

| pERK | *rd1* saline-treated | | | *rd1* TIMP1-treated | | |
| --- | --- | --- | --- | --- | --- | --- |
|  | Animal 1 | Animal 2 | Animal 3 | Animal 1 | Animal 2 | Animal 3 |
| 5 min | 100.62 | 100.61 | 100.43 | 97.40 | 98.99 | 90.24 |
| 1 hr | 113.19 | 106.78 | 100.18 | 160.09 | 150.36 | 132.94 |
| 6 hrs | 115.04 | 94.46 | 101.56 | 101.18 | 97.44 | 86.13 |

| pAKT | *rd1* saline-treated | | | *rd1* TIMP1-treated | | |
| --- | --- | --- | --- | --- | --- | --- |
|  | Animal 1 | Animal 2 | Animal 3 | Animal 1 | Animal 2 | Animal 3 |
| 5 min | 100.22 | 101.06 | 100.96 | 105.66 | 111.22 | 92.33 |
| 1 hr | 105.98 | 107.41 | 94.90 | 95.77 | 96.41 | 92.17 |
| 6 hrs | 93.79 | 90.44 | 93.37 | 95.57 | 105.99 | 87.32 |
