# Supplementary material for: Tissue inhibitor of metalloproteinases 1 enhances rod survival in the rd1 mouse retina
Source: PLoS One. 2018 May 9;13(5):e0197322. doi: 10.1371/journal.pone.0197322 (PMC5942829; doi:10.1371/journal.pone.0197322)
Supplement: S6 Table — Immunoblot analysis shows no detectable changes in expression of pERK1/2 and pAKT in the SB-3CT treated rd1 retina, compared to saline-treated rd1 retina. β-actin was used as a loading control to obtain relative pERK1/2 and pAKT expression (Fig 5D–5F). (DOCX) [file pone.0197322.s010.docx]

**S6 Table. Quantification of pERK and pAKT expression in saline-treated vs. SB-3CT-treated retina by immunoblot analysis.**

| pERK | *rd1* saline-treated | | | *rd1* SB-3CT-treated | | |
| --- | --- | --- | --- | --- | --- | --- |
|  | Animal 1 | Animal 2 | Animal 3 | Animal 1 | Animal 2 | Animal 3 |
| 5 min | 100.51 | 100.87 | 100.41 | 99.91 | 96.04 | 91.12 |
| 1 hr | 118.93 | 110.92 | 120.53 | 135.18 | 126.46 | 146.21 |
| 6 hrs | 138.73 | 145.72 | 117.27 | 147.50 | 152.08 | 111.85 |

| pAKT | *rd1* saline-treated | | | *rd1* SB-3CT-treated | | |
| --- | --- | --- | --- | --- | --- | --- |
|  | Animal 1 | Animal 2 | Animal 3 | Animal 1 | Animal 2 | Animal 3 |
| 5 min | 100.52 | 100.75 | 100.50 | 103.55 | 99.76 | 99.40 |
| 1 hr | 113.69 | 109.64 | 103.82 | 95.19 | 90.32 | 119.24 |
| 6 hrs | 135.83 | 133.21 | 110.96 | 153.82 | 142.59 | 116.93 |
