# Supplementary material for: Tissue inhibitor of metalloproteinases 1 enhances rod survival in the rd1 mouse retina
Source: PLoS One. 2018 May 9;13(5):e0197322. doi: 10.1371/journal.pone.0197322 (PMC5942829; doi:10.1371/journal.pone.0197322)
Supplement: S7 Table — Immunoblot analysis shows suppression of BAX from 5minutes to 48 hours after TIMP1 injection at P15. β-actin was used as a loading control to obtain relative BAX expression (Fig 6). (DOCX) [file pone.0197322.s011.docx]

| *Time* | *rd1* saline-treated | | | | | | *rd1* TIMP1-treated | | | | | |
| --- | --- | --- | --- | --- | --- | --- | --- | --- | --- | --- | --- | --- |
|  | Animal 1 | Animal 2 | Animal 3 | Animal 4 | Animal 5 | Animal 6 | Animal 1 | Animal 2 | Animal 3 | Animal 4 | Animal 5 | Animal 6 |
| 5 min | 100.85 | 106.10 | 107.15 | 100.76 | 98.35 | 99.11 | 85.66 | 87.60 | 87.36 | 89.32 | 84.65 | 86.85 |
| 1 hr | 129.88 | 118.06 | 118.86 | 126.67 | 119.14 | 121.25 | 79.71 | 87.21 | 89.68 | 78.58 | 88.14 | 89.04 |
| 6 hrs | 106.18 | 100.15 | 103.92 | 99.90 | 97.23 | 102.83 | 82.41 | 81.44 | 84.33 | 81.08 | 80.48 | 78.80 |
| 24 hrs | 181.82 | 143.01 | 162.85 | 176.43 | 151.27 | 165.75 | 89.41 | 91.09 | 87.03 | 88.79 | 92.43 | 87.14 |
| 48 hrs | 54.30 | 66.35 | 64.57 | 52.87 | 63.67 | 64.65 | 17.43 | 16.45 | 24.61 | 18.88 | 16.76 | 23.47 |

**S7 Table. Quantification of BAX expression in saline-treated vs. TIMP1-treated retinas by immunoblot analysis.**
