# Supplementary material for: Tissue inhibitor of metalloproteinases 1 enhances rod survival in the rd1 mouse retina
Source: PLoS One. 2018 May 9;13(5):e0197322. doi: 10.1371/journal.pone.0197322 (PMC5942829; doi:10.1371/journal.pone.0197322)
Supplement: S8 Table — Amplitudes of b-wave were measured from P60 and P90 normal, saline-treated and TIMP1 treated rd1 retinas (Fig 7). (DOCX) [file pone.0197322.s012.docx]

**S8 Table. The amplitudes of b-wave photopic ERGs in normal, saline-treated and TIMP1-treated retinas.**

|  | *rd1* saline-treated | | | | | *rd1* TIMP1-treated | | | | |
| --- | --- | --- | --- | --- | --- | --- | --- | --- | --- | --- |
|  | Animal 1 | Animal 2 | Animal 3 | Animal 4 | Animal 5 | Animal 1 | Animal 2 | Animal 3 | Animal 4 | Animal  5 |
| P60 | 17.0 | 25.0 | 12.0 | 16.0 | 24.0 | 25.0 | 18.0 | 29.0 | 27.0 | 33.0 |
| P90 | 18.5 | 18.0 | 15.0 | 26.0 | 16.0 | 23.0 | 26.0 | 22.0 | 28.0 | 28.0 |

|  | Normal | | | | |
| --- | --- | --- | --- | --- | --- |
|  | Animal 1 | Animal 2 | Animal 3 | Animal 4 | Animal 5 |
| P60 | 53.0 | 54.8 | 63.5 | 72.3 | 59.0 |
| P90 | 56.2 | 53.8 | 54.0 | 58.0 | 51.1 |
